# Treatment monitoring of colorectal cancer by integrated analysis of plasma concentration and sequencing of circulating tumor DNA

**DOI:** 10.1186/s12943-020-01273-8

**Published:** 2020-10-26

**Authors:** Yu-Min Yeh, Peng-Chan Lin, Chung-Ta Lee, Shang-Hung Chen, Bo-Wen Lin, Shao-Chieh Lin, Po-Chuan Chen, Ren-Hao Chan, Meng-Ru Shen

**Affiliations:** 1 Department of Internal Medicine, National Cheng Kung University Hospital, College of Medicine, National Cheng Kung University, Tainan, Taiwan; 2 Department of Pathology, National Cheng Kung University Hospital, College of Medicine, National Cheng Kung University, Tainan, Taiwan; 3 Department of Surgery, National Cheng Kung University Hospital, College of Medicine, National Cheng Kung University, Tainan, Taiwan; 4 Department of Pharmacology, National Cheng Kung University Hospital, College of Medicine, National Cheng Kung University, Tainan, Taiwan; 5 Department of Obstetrics and Gynecology, National Cheng Kung University Hospital, College of Medicine, National Cheng Kung University, No. 138 Sheng-Li Road, Tainan, 704 Taiwan

**Keywords:** Colorectal cancer, Circulating cell-free DNA, Cell-free DNA concentration, Molecular barcode, Next-generation sequencing

## Abstract

Circulating cell-free DNA (cfDNA) analysis is an important tool for cancer monitoring. The patient-specific mutations identified in colorectal cancer (CRC) tissues are usually used to design the cfDNA analysis. Despite high specificity in predicting relapse, the sensitivity in most studies is around 40–50%. To improve this weakness, we designed a cfDNA panel according to the CRC genomic landscape and recurrent-specific mutations. The pathological variants in cfDNA samples from 60 CRC patients were studied by a next-generation sequencing (NGS) method incorporating the dual molecular barcode. Interestingly, patients in the disease positive group had a significantly higher cfDNA concentration than those in the disease negative group. Based on receiver operating characteristic analysis, the cfDNA concentration of 7 ng/mL was selected into the analytical workflow. The sensitivity in determining the disease status was 72.4%, which represented a considerable improvement on prior studies, and the specificity remained high at 80.6%. Compared to standard imaging and laboratory studies, earlier detection of residual disease and clinical benefits were shown on two cases by this cfDNA assay. We conclude this integrative framework of cfDNA analytical pipeline with a satisfactory sensitivity and specificity could be used in postoperative CRC surveillance.

**Supplementary Information:**

**Supplementary information** accompanies this paper at 10.1186/s12943-020-01273-8.

## Main text

The standard care for CRC patients without distant metastasis is surgical resection of the primary tumor with en bloc removal of the regional lymph nodes. Despite the aggressive use of adjuvant FOLFOX chemotherapy, recurrence develops in a third of patients with stage III or high-risk stage II disease. Moreover, FOLFOX chemotherapy causes a long-lasting neurotoxicity, negatively impacting the quality of life of survivors [1, 2]. The development of biomarkers to identify CRC patients who truly have residual disease will allow patients to be treated with appropriate adjuvant chemotherapy and avoid unnecessary treatment-related toxicity in the adjuvant setting.

Circulating cfDNA are short fragmented DNAs which are detectable in plasma [3]. Several studies selected the patient-specific mutations identified in the tumor tissues for cfDNA analysis of blood sample and proved the use for postoperative surveillance with different sequencing technologies [4–6]. Overall, the results from these studies [4–6] showed that detection of mutations in cfDNA was strongly associated with inferior recurrence-free survival. The specificity in predicting the relapse was high (96–100%); however, the sensitivity in predicting relapse was around 40–50%.

To improve the unsatisfactory sensitivity of cfDNA test in predicting tumor recurrence, the important and recurrence-specific CRC mutations identified in our previous study [7] were used to design a cfDNA panel, and a sequencing method incorporating molecular tags was used to detect the low-frequency cfDNA mutations. Moreover, the cfDNA concentration was integrated into the analytical pipeline. This cfDNA analytical workflow showed the satisfactory sensitivity and specificity of determining the disease status were 72.4 and 80.6%, respectively.

## Results

### Clinical cohort

Sixty-nine genes were included in the cfDNA panel (Supplementary Table S1), which was tested in 60 histologically confirmed CRC patients, including 31 patients with no clinical evidence of disease (disease negative group), and 29 patients with clinical evidence of disease (disease positive group). Patients with and without clinical evidence of disease were determined by the results of the most recent imaging studies. The clinicopathological and molecular characteristics were similar between the two groups, except for the stage at initial diagnosis (Supplementary Table S2). cfDNA analysis was tested three times at intervals of 3–6 months for each patient. Because of the loss to follow-up and disease progression leading to death, the results of the 2nd and 3rd cfDNA analysis were available in 54 and 44 patients, respectively (Supplementary Fig. S1).

### cfDNA concentrations

**Fig. 1:**
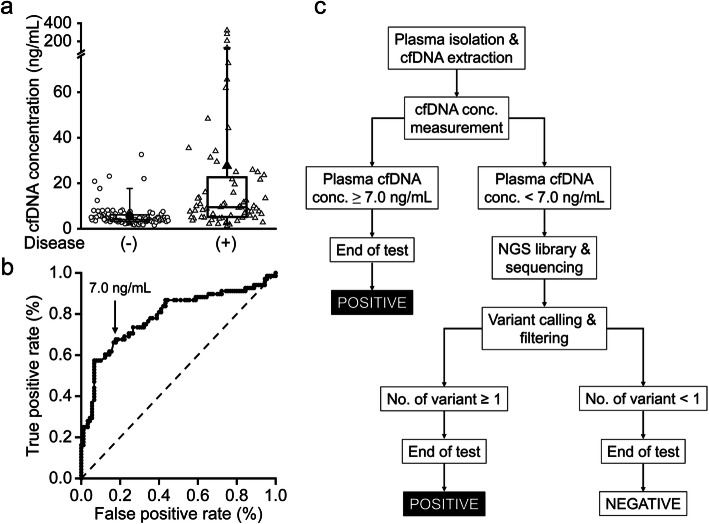
Proposed workflow of cfDNA analysis for treatment monitoring of CRC patients. **a** Boxplot displaying the cfDNA levels in plasma collected from patients in the disease negative (open circle; *n* = 90) and disease positive (open triangle; *n* = 68) groups at three different time points. The horizontal box boundaries and midline represent the sample quartiles, while the solid circle and triangle indicate the mean of the cfDNA concentrations. The upper and lower whisker denotes the 95th and 5th percentiles, respectively. **b** The receiver operating characteristics (ROC) curve of cfDNA concentration in determining the presence or absence of disease. The points in the ROC curve indicate different cfDNA concentrations with corresponding true and false positive rates, from which 7.0 ng/mL was chosen as the cut-off value for cfDNA concentration. The dotted line is the line of no-discrimination. **c** The cfDNA concentrations and sequencing results were integrated together in the analytical workflow. For patients with cfDNA concentration ≥ 7.0 ng/mL, the cfDNA test was defined as positive, and for patients with cfDNA concentration < 7.0 ng/mL, the detection of at least one cfDNA variant was required to define a positive cfDNA test

A total of 158 plasma samples were collected. The cfDNA concentrations of these samples are shown in Fig. 1a. In the disease negative group, the majority (84 of 90) of the cfDNA concentrations were less than 10 ng/mL, and the median cfDNA concentration was 4.09 ng/mL. In contrast, the cfDNA concentration in the disease positive group varied from 1.180 to 321 ng/mL, and the median level was 9.47 ng/mL. Patients in the disease positive group had a significantly higher cfDNA concentration than those in the disease negative group. ROC analysis was performed to determine an optimal threshold, demonstrating that 7.0 ng/mL had the best ability to differentiate two groups (Fig. 1b).

### Pathological cfDNA mutations

The library preparation method utilizing molecular barcodes was adopted by the cfDNA assay in order to detect low-frequency variants and improve the sensitivity. The analytical pipeline was shown in Supplementary Fig. S2 and the details of variant calling, annotation and filtering were provided in Supplementary Materials and Methods. When variants with an allele frequency (AF) ≥ 0.1% were used to determine the variant detected in cfDNA and plasma samples were defined as positive, the cfDNA assay showed a very good sensitivity (0.828); however, the specificity was only 0.484, and the accuracy was 0.65 (Supplementary Table S3). When the AF cut-off was shifted from 0.1 to 0.2, 0.3, or 0.5, the sensitivity mildly decreased from 0.828 to 0.793, 0.724, or 0.724 respectively. Concurrently, the improvement of the specificity was observed (from 0.484 to 0.742, 0.806, and 0.806). The best accuracy was 0.767 with the Youden index of 0.53, which was detected at cut-off levels of 0.3 and 0.5%; thus, 0.5% was chosen for subsequent analyses.

**Fig. 2:**
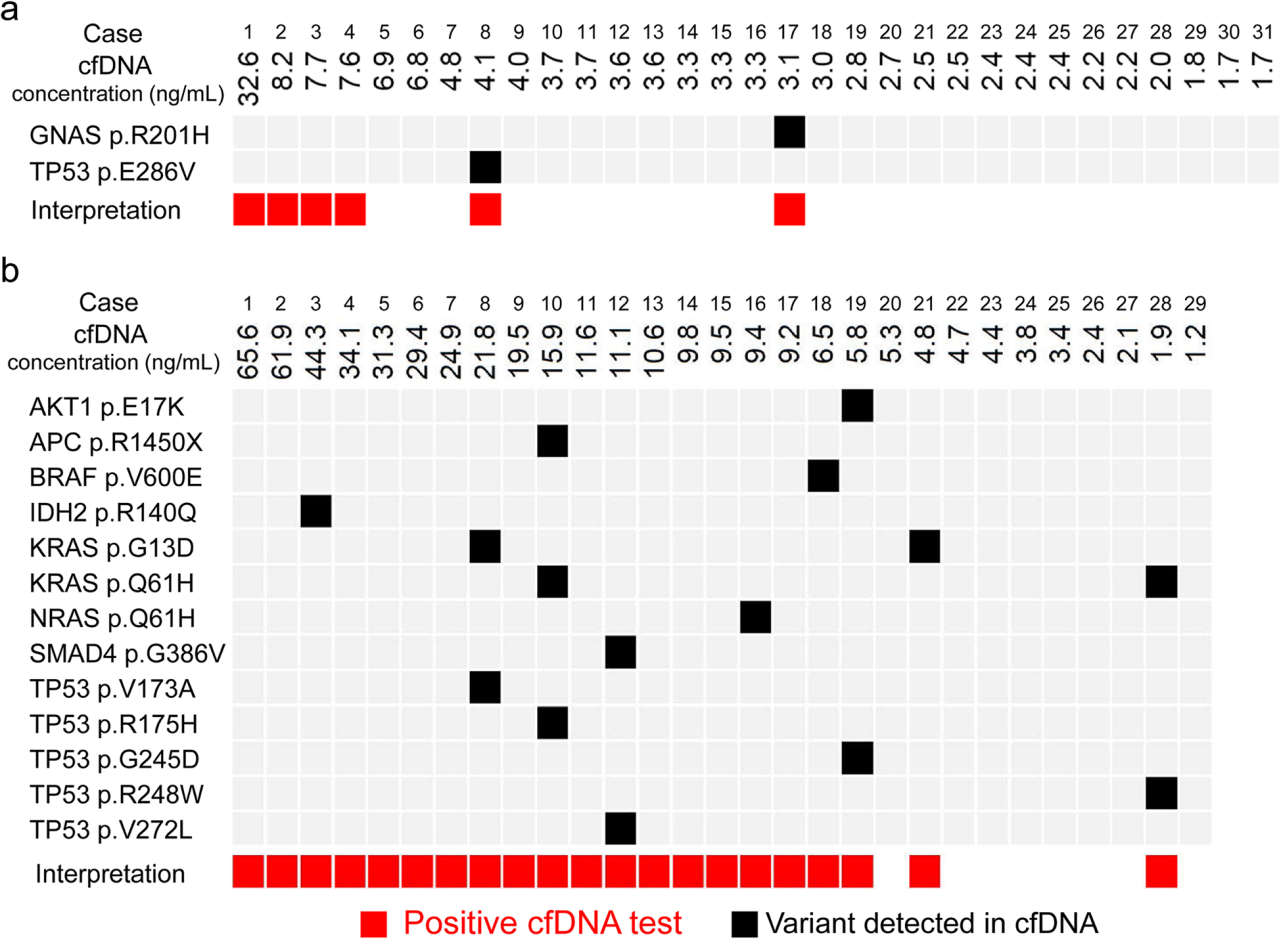
Results of integrated cfDNA analysis. The final result of cfDNA analysis integrated from the cfDNA concentration and variants detected in the cfDNA samples of the disease negative (**a**) and positive (**b**) groups are shown. A concentration of cfDNA ≥7.0 ng/mL, and/or at least 1 variant detected in plasma samples was used to define a positive cfDNA test. The black solid squares indicate the variants detected in cfDNA samples, and the red solid squares indicate that the result of integrated cfDNA analysis is positive

We proposed a workflow by integrating the concentration and pathological variants of cfDNA (Fig. 1c). When the cfDNA concentration was ≥7.0 ng/mL, the test was defined as positive. In patients with a cfDNA concentration < 7.0 ng/mL, one or more variants detected in plasma samples was required to define as positive. Figure 2 showed the result of the 1st cfDNA analysis using the cfDNA panel in 60 CRC patients. In the disease negative group (*N* = 31), 4 patients (Cases 1–4) had a cfDNA concentration > 7.0 ng/mL, with no mutation detected in cfDNA samples. Two patients (Cases 8 and 17) had variants detected in cfDNA samples, but the cfDNA concentration of these patients was < 7.0 ng/mL (Fig. 2a). According to the proposed workflow, these patients in the disease negative group had positive cfDNA analysis. Tumor recurrence was detected in one (Case 4) of the 6 patients 4 months after the cfDNA analysis.

In the disease positive group, 21 of 29 patients showed positive cfDNA results; 17 patients (Cases 1–17) had a cfDNA concentration ≥ 7.0 ng/mL (Fig. 2b). Among the 12 patients with a cfDNA concentration < 7.0 ng/mL, variants were detected in 4 patients (Cases 18, 19, 21, and 28). In the disease positive group, the variants were detected most frequently in *TP53* (*n* = 5) and *KRAS* (*n* = 4) (Supplementary Table S4).

### Performance in 2nd and 3rd cfDNA analysis

The cfDNA analysis was repeated at intervals of 3–6 months. When the results of the 2nd and 3rd cfDNA analysis were pooled, the sensitivity, specificity, and accuracy were 0.821, 0.729, and 0.765, similar to those observed in the 1st cfDNA analysis (Supplementary Table S5). *TP53* and *KRAS* were still the most common genes with variants detected in the cfDNA (Supplementary Table S6 and S7).

### Clinical benefits

**Fig. 3:**
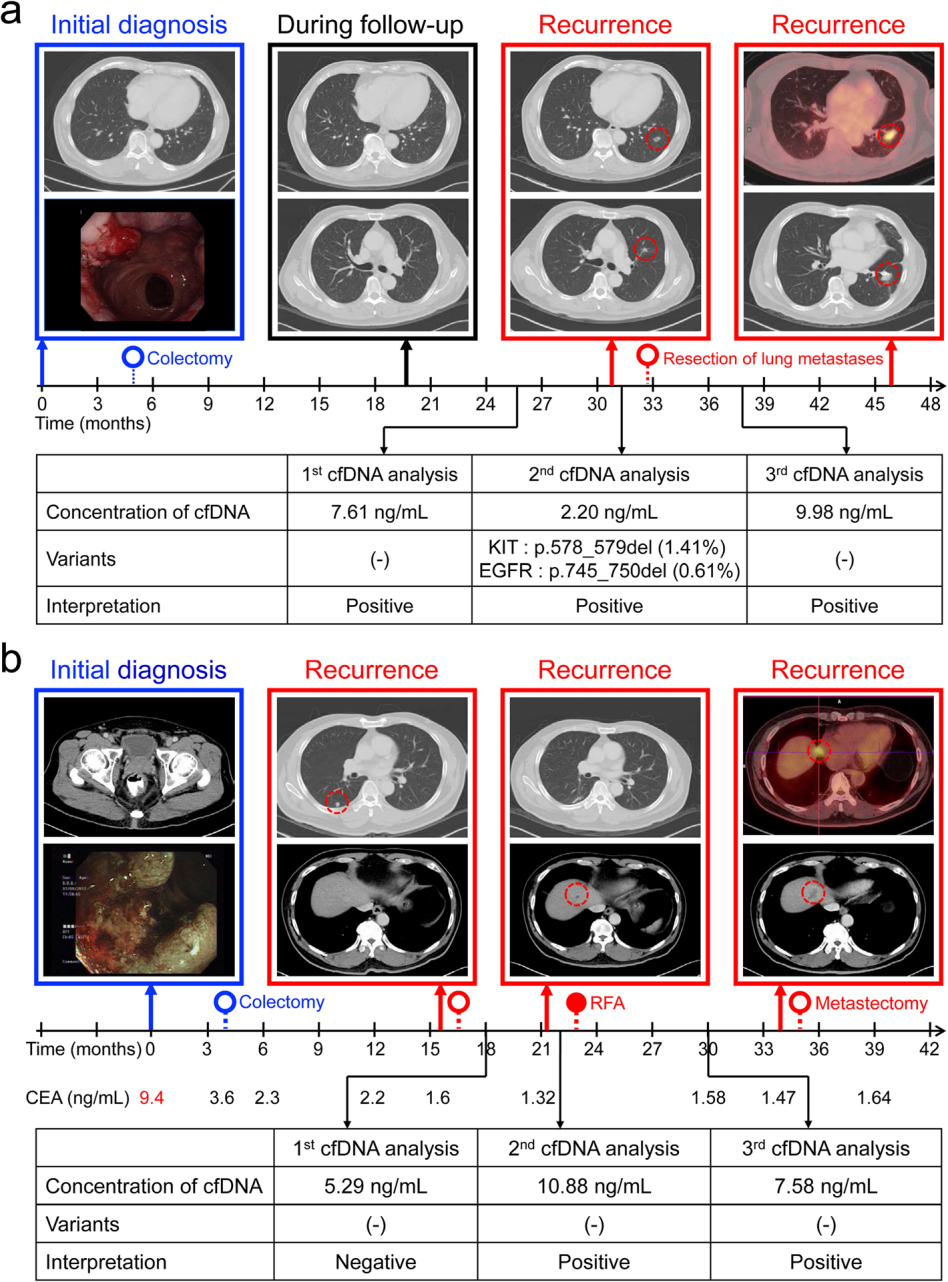
Clinical benefits of the cfDNA assay in two CRC patients. **a** This patient had a rectal adenocarcinoma, which was treated with pre-operative concurrent chemoradiotherapy followed by a rectum colectomy and post-operative adjuvant chemotherapy. Recurrence with pulmonary metastases was detected 2 years after surgery of the primary tumor. The patient underwent resection of the pulmonary metastases with curative intent, and was treated with systemic chemotherapy for 3 months. The result of the 3rd cfDNA analysis was positive, although the imaging study at that time did not detect any recurrence. Later imaging revealed recurrent tumors around the surgical sutures of the left upper and left lower lobe. **b** This patient had pathological stage III rectal adenocarcinoma, which was treated with pre-operative concurrent chemoradiotherapy, rectum colectomy, and post-operative adjuvant FOLFOX chemotherapy for 6 months. Recurrence with pulmonary metastasis was detected 1 year after surgery of the primary tumor. The patient underwent complete resection of the pulmonary metastasis, and received salvage chemotherapy with bevacizumab in combination with FOLFIRI. Five months after the metastectomy, the imaging study revealed a new liver metastasis, which was treated with percutaneous radiofrequency ablation (RFA). Following RFA, the cfDNA concentration remained high, which indicated the presence of disease; however, the imaging study at that time did not detect any recurrence. A later CT scan revealed a recurrent tumor in the liver, adjacent to the previous ablation site

The routine post-treatment surveillance of CRC patients include history, physical examination, carcinoembryonic antigen (CEA) test, and computed tomography (CT) scan. Based on the proposed workflow, two cases showed the clinical benefit of cfDNA assay. Figure 3a outlines the case of a 70-year-old male patient with stage IIIA rectal cancer. After the surgery, lung metastases were detected by CT scan 30 months after initial diagnosis. The patient received resection of the lung lesions with curative intent. The cfDNA test of the 3rd analysis at 38 months was positive based on the high cfDNA concentration (9.98 ng/mL), but there was no evidence of recurrence by CT scan. However, 8 months later, CT scan showed recurrent tumors around the surgical sutures of the left lung, confirmed by positron emission tomography (PET) scan. Figure 3b highlights another case in the disease positive group. The patient was diagnosed with clinical stage III rectal cancer and the CEA level was 9.4 ng/mL at initial diagnosis. After the surgery, the CEA level decreased to 2.3 ng/mL and remained normal in all subsequent tests. During the follow-up, lung and liver metastases were detected. The patient received the surgical resection of lung metastases and percutaneous radiofrequency ablation for liver metastasis. The CT scans at 27 and 30 months did not detect any recurrence but the cfDNA concentration of the 3rd analysis at 30 months was still high (7.58 ng/mL), indicating a positive test for the disease status. A recurrent tumor in the liver, adjacent to the previous ablation site, was demonstrated by the CT scan 4 months after the 3rd cfDNA analysis. Moreover, the *BRAF* V600E mutation detected in Case 18 showing a response to the treatment combining a BRAF, MEK and EGFR inhibitor and the disease progression accompanied by the increase of cfDNA concentration observed in many cases in the disease positive group also suggest the potential utility of integrated cfDNA analysis in prediction and monitoring treatment response in advanced colorectal cancer.

## Discussion

This is the first study to integrate the cfDNA concentration into the workflow of cfDNA analysis for monitoring the disease status of CRC patients. The concept of applying cfDNA concentration for postoperative surveillance of CRC patients is supported by a recent study [8]. The tumor heterogeneity and clonal evolution might be the reasons explaining the low sensitivity in prior studies using the patient-specific cfDNA analysis [9]. Tracking multiple mutations across a wide range of genes without personalization is a solution to overcome this limitation [10] and the results of this study are in agreement with this strategy.

## Conclusions

This cfDNA analytical pipeline, integrating the cfDNA concentration and pathological variants detected by an ultra-sensitive sequencing technique, shows a satisfactory sensitivity in postoperative CRC surveillance.

## Supplementary Information

**Additional file 1:.** Supplementary materials and methods.

**Additional file 2: Supplementary Table S1.** Genes included in the cfDNA assay**. Supplementary Table S2.** Clinical characteristics of CRC patients in the disease negative and positive group. **Supplementary Table S3.** The performance of the cfDNA test when applying different cutoff levels of allele frequency (AF). **Supplementary Table S4.** Variants detected in 1st cfDNA analysis. **Supplementary Table S5.** Performance of the cfDNA assay in 2nd and 3rd cfDNA analysis. **Supplementary Table S6.** Variants detected in 2nd cfDNA analysis. **Supplementary Table S7.** Variants detected in 3rd cfDNA analysis.

**Additional file 3: Supplementary Figure S1.** The number and timing of cfDNA analysis in 60 CRC patients. **Supplementary Figure S2.** Analytical pipeline of the cfDNA test.

## Data Availability

All data generated during this study are included in this published article and its supplementary information files.

## Abbreviations

AF: Allele frequency
cfDNA: Cell-free DNA
CEA: Carcinoembryonic antigen
CRC: Colorectal cancer
CT: Computed tomography
FOLFIRI: 5-fluorouracil, leucovorin, irinotecan
FOLFOX: 5-fluorouracil, leucovorin, oxaliplatin
NGS: Next generation sequencing
PET: Positron emission tomography
RFA: Radiofrequency ablation
ROC: Receiver operating characteristic
