# Supplementary material for: Treatment monitoring of colorectal cancer by integrated analysis of plasma concentration and sequencing of circulating tumor DNA
Source: Mol Cancer. 2020 Oct 26;19:150. doi: 10.1186/s12943-020-01273-8 (PMC7586655; doi:10.1186/s12943-020-01273-8)
Supplement: Supplementary file 1 — Additional file 1:. Supplementary materials and methods. [file 12943_2020_1273_MOESM1_ESM.docx]

**Supplementary Materials and Methods**

**Study design**

A total of 60 histologically confirmed CRC patients, including 29 patients with clinical evidence of disease (disease positive group), and 31 patients without clinical evidence of disease (disease negative group), were prospectively enrolled from National Cheng Kung University Hospital (NCKUH). Patients with and without clinical evidence of disease were determined by the results of the most recent imaging studies. The clinicopathological and molecular characteristics, including the age of onset; sex; histology; stage at initial diagnosis; site of primary tumor; status of *KRAS*, *NRAS*, *BRAF* mutation; *HER2* overexpression; and MMR/MSI, were obtained from the patients’ medical records. For cfDNA analysis, 10 mL of peripheral blood was collected three times at intervals of 3–6 months. The results of cfDNA analysis were compared with the clinical status. The performance of the cfDNA test to determine the presence or absence of disease, including the sensitivity, specificity, and accuracy, was calculated. This study was approved by the Institutional Review Board of NCKUH (A-ER-108-033), and written informed consent was provided by the participants before enrollment.

**cfDNA assay**

The important and recurrence-specific CRC mutations identified in our previous study, together with well-known mutated genes were used to design the cfDNA panel (1). Briefly, patients with pathological stage III CRC were prospectively enrolled from the NCKUH clinical study that had been investigating the chemotherapy-induced peripheral neuropathy since Jan 2015 (2). Deep targeted sequencing was performed using the Oncomine Comprehensive Assay, version 1 (Thermo Fisher Scientific) in order to identify important mutations in primary and recurrent tumor samples of this CRC cohort. A total of 434 somatic mutations across 48 genes were detected in the 98 primary tumor samples, and 10 mutated genes were identified in the 9 recurrent tumors. These important and recurrence-specific CRC mutations, together with frequently detected mutations in multiple cancer types were used to design this cfDNA panel. A total of 69 genes were included in this cfDNA panel, and the details of the gene list are provided in Supplementary Table S1.

**Extraction of cfDNA**

After 10 mL of peripheral blood was drawn, the sample was collected in a PAXgene Blood ccfDNA tube and shipped to the lab within 24 hours at room temperature (15°C–25°C). After centrifugation, the plasma was collected and the cfDNA was extracted using the Applied Biosystems™ MagMAX™ Cell-Free DNA Isolation Kit. The concentration of the cfDNA was determined by the Invitrogen™ Qubit™ Fluorometer with the Qubit™ dsDNA High Sensitivity Assay (Thermo Fisher Scientific).

**Ion AmpliSeq HD library preparation**

Target regions of cfDNA were amplified using the customer designed Ion AmpliSeq HD primer containing 153 paired oligomers. Multiplex PCR and library construction were performed according to the Ion AmpliSeq HD Library Kit User Guide (Thermo Fisher Scientific). Briefly, 20 ng cfDNA was used for Multiplex PCR. First, the target regions were amplified by PCR for three cycles, the PCR products were partially digested by SUPA reagent, and the HD library was amplified with the Ion AmpliSeq HD Dual Barcode kit. The library concentration was measured by Qubit dsDNA High Sensitivity Assay and Ion Library Quantitation Kit.

**Next generation sequencing**

Each library was diluted to 100 pM and pooled with other libraries of the same amount for one sequence reaction. Template preparation and chip loading were performed with the Ion 540 Kit-Chef (Thermo Fisher Scientific) in the Ion Chef System. The Ion 540 Kit-Chef was used with the Ion S5™ XL sequencer (Thermo Fisher Scientific) as described in the Ion 540™ Kit - Chef User Guide.

**Bioinformatics and statistical analysis**

Data quality control, alignment, variant calling, and limit of detection (LOD) calculation were conducted using a locked data analysis pipeline provided by Thermo Fisher Scientific. The workflows “AmpliSeq HD for Liquid Biopsy w2.2 – DNA, and Fusions (Single Library) - Single Sample_noCNV” in Ion Reporter version 5.10 (Thermo Fisher Scientific) were used. This analysis pipeline utilized hg19 as the reference genome, and provided single nucleotide variants (SNVs) and insertion-deletion mutations (Indels) callings with allele frequencies (AF) as low as 0.05%. Variant annotation was performed using ANNOVAR version 2018Apr16. Variants in vcf files were retained if they satisfied the following two criteria: 1) The AF was equal to or higher than the defined threshold, and 2) the clinvar (version 2019Mar05) annotation was pathogenic, likely pathogenic, or drug response. The result of the cfDNA test was reported as positive if any variant was observed. Unpaired *t-*test and Fisher’s exact test were used to compare differences between the disease negative and disease positive groups. Receiver operating characteristic (ROC) analysis was used to determine the optimal cut-off of cfDNA concentration for predicting the patients in the disease positive group and those in the disease negative group.

**Reference**

1. Lin PC, Yeh YM, Wu PY, Hsu KF, Chang JY, Shen MR. Germline susceptibility variants impact clinical outcome and therapeutic strategies for stage III colorectal cancer. Sci Rep. 2019;9(1):3931.

2. Huang HW, Wu PY, Su PF, Li CI, Yeh YM, Lin PC, et al. A Simplified Diagnostic Classification Scheme of Chemotherapy-Induced Peripheral Neuropathy. Dis Markers. 2020;2020:3402108.
