# Supplementary material for: Treatment monitoring of colorectal cancer by integrated analysis of plasma concentration and sequencing of circulating tumor DNA
Source: Mol Cancer. 2020 Oct 26;19:150. doi: 10.1186/s12943-020-01273-8 (PMC7586655; doi:10.1186/s12943-020-01273-8)
Supplement: Supplementary file 2 — Additional file 2: Supplementary Table S1. Genes included in the cfDNA assay. Supplementary Table S2. Clinical characteristics of CRC patients in the disease negative and positive group. Supplementary Table S3. The performance of the cfDNA test when applying different cutoff levels of allele frequency (AF). Supplementary Table S4. Variants detected in 1st cfDNA analysis. Supplementary Table S5. Performance of the cfDNA assay in 2nd and 3rd cfDNA analysis. Supplementary Table S6. Variants detected in 2nd cfDNA analysis. Supplementary Table S7. Variants detected in 3rd cfDNA analysis. [file 12943_2020_1273_MOESM2_ESM.zip › Supplementary Table S1.docx]

**Supplementary Table S1.** Genes included in the cfDNA assay

|  | Gene names |  | Gene names |  | Gene names |
| --- | --- | --- | --- | --- | --- |
| 1 | AKT1 | 24 | ESR1 | 47 | NF1 |
| 2 | ALK | 25 | FBXW7 | 48 | NF2 |
| 3 | APC | 26 | FGFR1 | 49 | NOTCH1 |
| 4 | AR | 27 | FGFR2 | 50 | NRAS |
| 5 | ARAF | 28 | FGFR3 | 51 | NTRK1 |
| 6 | ATM | 29 | FGFR4 | 52 | NTRK3 |
| 7 | BRAF | 30 | FLT3 | 53 | PDGFRA |
| 8 | BRCA1 | 31 | GNA11 | 54 | PIK3CA |
| 9 | BRCA2 | 32 | GNAQ | 55 | PIK3R1 |
| 10 | CCND1 | 33 | GNAS | 56 | PTCH1 |
| 11 | CCND2 | 34 | HRAS | 57 | PTEN |
| 12 | CCND3 | 35 | IDH1 | 58 | RAC1 |
| 13 | CDH1 | 36 | IDH2 | 59 | RAF1 |
| 14 | CDK4 | 37 | JAK3 | 60 | RB1 |
| 15 | CDK6 | 38 | KIT | 61 | RET |
| 16 | CHEK2 | 39 | KRAS | 62 | ROS1 |
| 17 | CSF1R | 40 | MAP2K1 | 63 | SFSB1 |
| 18 | CTNNB1 | 41 | MAP2K2 | 64 | SMAD4 |
| 19 | DDR2 | 42 | MCL1 | 65 | SMARCB1 |
| 20 | DNMT3A | 43 | MET | 66 | SMO |
| 21 | EGFR | 44 | MLH1 | 67 | TP53 |
| 22 | ERBB2 | 45 | MTOR | 68 | TSC1 |
| 23 | ERBB3 | 46 | MYC | 69 | VHL |
